# Harnessing cytokine-induced killer cells to accelerate diabetic wound healing: an approach to regulating post-traumatic inflammation

**DOI:** 10.1093/rb/rbad116

**Published:** 2024-01-09

**Authors:** Yixi Yang, Cheng Zhang, Yuan Jiang, Yijun He, Jiawei Cai, Lin Liang, Zhaohuan Chen, Sicheng Pan, Chu Hua, Keke Wu, Le Wang, Zhiyong Zhang

**Affiliations:** Translational Research Centre of Regenerative Medicine and 3D Printing of Guangzhou Medical University, Guangdong Province Engineering Research Center for Biomedical Engineering, State Key Laboratory of Respiratory Disease, Department of Orthopaedic Surgery, Medical Technology and Related Equipment Research for Spinal Injury Treatment, City Key Laboratory, The Third Affiliated Hospital of Guangzhou Medical University, School of Biomedical Engineering, Guangzhou Medical University, Guangzhou, Guangdong 510150, P. R. China; Translational Research Centre of Regenerative Medicine and 3D Printing of Guangzhou Medical University, Guangdong Province Engineering Research Center for Biomedical Engineering, State Key Laboratory of Respiratory Disease, Department of Orthopaedic Surgery, Medical Technology and Related Equipment Research for Spinal Injury Treatment, City Key Laboratory, The Third Affiliated Hospital of Guangzhou Medical University, School of Biomedical Engineering, Guangzhou Medical University, Guangzhou, Guangdong 510150, P. R. China; Translational Research Centre of Regenerative Medicine and 3D Printing of Guangzhou Medical University, Guangdong Province Engineering Research Center for Biomedical Engineering, State Key Laboratory of Respiratory Disease, Department of Orthopaedic Surgery, Medical Technology and Related Equipment Research for Spinal Injury Treatment, City Key Laboratory, The Third Affiliated Hospital of Guangzhou Medical University, School of Biomedical Engineering, Guangzhou Medical University, Guangzhou, Guangdong 510150, P. R. China; Department of Osteoarthropathy and Sports Medicine, Panyu Central Hospital, Guangzhou 511400, P. R. China; Translational Research Centre of Regenerative Medicine and 3D Printing of Guangzhou Medical University, Guangdong Province Engineering Research Center for Biomedical Engineering, State Key Laboratory of Respiratory Disease, Department of Orthopaedic Surgery, Medical Technology and Related Equipment Research for Spinal Injury Treatment, City Key Laboratory, The Third Affiliated Hospital of Guangzhou Medical University, School of Biomedical Engineering, Guangzhou Medical University, Guangzhou, Guangdong 510150, P. R. China; Translational Research Centre of Regenerative Medicine and 3D Printing of Guangzhou Medical University, Guangdong Province Engineering Research Center for Biomedical Engineering, State Key Laboratory of Respiratory Disease, Department of Orthopaedic Surgery, Medical Technology and Related Equipment Research for Spinal Injury Treatment, City Key Laboratory, The Third Affiliated Hospital of Guangzhou Medical University, School of Biomedical Engineering, Guangzhou Medical University, Guangzhou, Guangdong 510150, P. R. China; Translational Research Centre of Regenerative Medicine and 3D Printing of Guangzhou Medical University, Guangdong Province Engineering Research Center for Biomedical Engineering, State Key Laboratory of Respiratory Disease, Department of Orthopaedic Surgery, Medical Technology and Related Equipment Research for Spinal Injury Treatment, City Key Laboratory, The Third Affiliated Hospital of Guangzhou Medical University, School of Biomedical Engineering, Guangzhou Medical University, Guangzhou, Guangdong 510150, P. R. China; Translational Research Centre of Regenerative Medicine and 3D Printing of Guangzhou Medical University, Guangdong Province Engineering Research Center for Biomedical Engineering, State Key Laboratory of Respiratory Disease, Department of Orthopaedic Surgery, Medical Technology and Related Equipment Research for Spinal Injury Treatment, City Key Laboratory, The Third Affiliated Hospital of Guangzhou Medical University, School of Biomedical Engineering, Guangzhou Medical University, Guangzhou, Guangdong 510150, P. R. China; Translational Research Centre of Regenerative Medicine and 3D Printing of Guangzhou Medical University, Guangdong Province Engineering Research Center for Biomedical Engineering, State Key Laboratory of Respiratory Disease, Department of Orthopaedic Surgery, Medical Technology and Related Equipment Research for Spinal Injury Treatment, City Key Laboratory, The Third Affiliated Hospital of Guangzhou Medical University, School of Biomedical Engineering, Guangzhou Medical University, Guangzhou, Guangdong 510150, P. R. China; Translational Research Centre of Regenerative Medicine and 3D Printing of Guangzhou Medical University, Guangdong Province Engineering Research Center for Biomedical Engineering, State Key Laboratory of Respiratory Disease, Department of Orthopaedic Surgery, Medical Technology and Related Equipment Research for Spinal Injury Treatment, City Key Laboratory, The Third Affiliated Hospital of Guangzhou Medical University, School of Biomedical Engineering, Guangzhou Medical University, Guangzhou, Guangdong 510150, P. R. China; Translational Research Centre of Regenerative Medicine and 3D Printing of Guangzhou Medical University, Guangdong Province Engineering Research Center for Biomedical Engineering, State Key Laboratory of Respiratory Disease, Department of Orthopaedic Surgery, Medical Technology and Related Equipment Research for Spinal Injury Treatment, City Key Laboratory, The Third Affiliated Hospital of Guangzhou Medical University, School of Biomedical Engineering, Guangzhou Medical University, Guangzhou, Guangdong 510150, P. R. China; Translational Research Centre of Regenerative Medicine and 3D Printing of Guangzhou Medical University, Guangdong Province Engineering Research Center for Biomedical Engineering, State Key Laboratory of Respiratory Disease, Department of Orthopaedic Surgery, Medical Technology and Related Equipment Research for Spinal Injury Treatment, City Key Laboratory, The Third Affiliated Hospital of Guangzhou Medical University, School of Biomedical Engineering, Guangzhou Medical University, Guangzhou, Guangdong 510150, P. R. China

**Keywords:** macrophage, diabetic wound, post-traumatic inflammation, cytokine-induced killer cells, vascularization

## Abstract

Impaired immunohomeostasis in diabetic wounds prolongs inflammation and cytokine dysfunction, thus, delaying or preventing wound-surface healing. Extensive clinical studies have been conducted on cytokine-induced killer (CIK) cells recently, as they can be easily proliferated using a straightforward, inexpensive protocol. Therefore, the function of CIK cells in regulating inflammatory environments has been drawing attention for clinical management. Throughout the current investigation, we discovered the regenerative capacity of these cells in the challenging environment of wounds that heal poorly due to diabetes. We demonstrated that the intravenous injection of CIK cells can re-establish a proregenerative inflammatory microenvironment, promote vascularization and, ultimately, accelerate skin healing in diabetic mice. The results indicated that CIK cell treatment affects macrophage polarization and restores the function of regenerative cells under hyperglycemic conditions. This novel cellular therapy offers a promising intervention for clinical applications through specific inflammatory regulation functions.

## Graphical Abstract

**Graphical Abstract:**
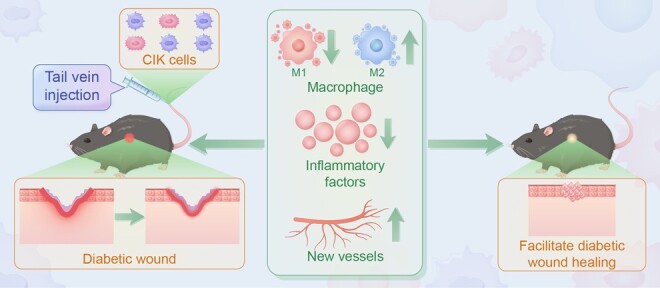

## Introduction 

The healing of diabetic wounds requires a complex series of histological and cell biological processes, including inflammation, coagulation, proliferation and remodeling [1]. This pathogenesis complicates the treatment of diabetic wounds, thus, degrading patient health [2]. Post-traumatic homeostasis in diabetes leads to various cytokine abnormalities, persistent cell dysfunction and apoptosis [3]. Compromised immunohomeostasis is characterized by persistent, excessive chronic inflammation, which is a hallmark of delayed diabetic wound healing [4]. Tissue microenvironmental homeostasis affects the ability of the traumatized tissue to regenerate, and the intrinsic cytokine complex is critical for the cytokine-mediated pattern of the inflammation stage, playing a pivotal role in re-establishing homeostasis [5].

The polarization morphology of macrophages is strongly connected to their biological functionality and serves as a crucial regulatory factor in the inflammatory change pattern of the tissue microenvironment in diabetic wound healing [6]. Macrophages can rapidly switch their phenotypes when the tissue microenvironment changes. They can be induced into a pro-inflammatory M1 form using gamma-interferon (IFN-γ) and lipopolysaccharide (LPS) [7] and can be activated into an M2 form (with an anti-inflammatory function) using interleukin-4 (IL-4), interleukin-10 (IL-10) and certain antioxidants [8]. However, an imbalance between the macrophages of M1 and M2 within the body can occur under physiological (ontogenetic and gestational) or pathological (acute and chronic inflammation, atherosclerosis and cancer) conditions [9]. While the mechanism causing the retardation of tissue repair in the diabetic microenvironment remains undefined, many studies have demonstrated that immunomodulation is indispensable to neovascularization, where macrophages are vital in the immune response [10]. The impaired microenvironmental homeostasis resulting from the build-up of advanced glycation end products (AGEs) through diabetic wounds is another important cause of imbalanced M1 and M2 macrophages [11]. Therefore, an active and precise regulation of macrophage polarization in AGE environments can restore local microenvironmental homeostasis.

Cytokine-induced killer (CIK) cells have been utilized in studies focusing on the treatment of tumor cells, such as renal cell carcinoma [12], as well as in research related to purging the marrow for autologous bone marrow transplantation [13]. The population of CIK cells is heterogeneous, including CD3^+^CD56^+^ natural killer T (NKT), CD3^+^CD56^−^ T and CD3^−^CD56^+^ natural killer (NK) cells [14]. NK cells respond efficiently to the inflammatory stimulation caused by tissue damage and regulate inflammatory environments [15]. Certain scientific discoveries have demonstrated that NK cells can reduce local immune responses and relieve inflammation by killing a portion of immune cells, including autologous stimulated T cells, dendritic cells, and monocytes [16]. In addition, NKT cells’ coordinating role contributes to the evolution from inflammation to tissue restoration after trauma, thus, promoting wound repair [17]. In an inflammatory environment, these cells can be activated to produce anti-inflammatory cytokines, including IL-4 and IL-10, thus, changing the phenotype of macrophages [18]. The absence of NKT cells or the blocking of their activation can reduce the amount of IL-4 and IL-10, delay the transition of macrophage phenotypes and interrupt revascularization and repair [19]. Furthermore, these cells can promote collagen deposition, myofibroblast differentiation and angiogenesis by inducing macrophages and fibroblasts to produce various cytokines [20]. In addition, the safe injection of CIK cells in patients has been proved by numerous clinical trials [21].

We previously reported that the remodeling of inherent post-traumatic inflammatory patterning impacts homeostatic restoration [22]. Given the significant effect of CIK cells on inflammatory regulation and wound repair, we hypothesized that they can enhance the healing of diabetic wounds by remodeling the disrupted inflammatory microenvironment after trauma. Within the context of our investigation, we removed dorsal skin from the backs of streptozotocin (STZ)-induced mice with type 1 diabetes to create a refractory wound model manifested with excessive inflammation [23]. The results of CIK cell injection proved that this therapy mediates specific inflammatory patterning in diabetic wounds and promotes skin regeneration. This accurate and proactive immunoregulation strategy can restore balance in diabetic wounds, modify macrophage polarization tendencies and enhance cell proliferation and differentiation by reactivating cell growth factors. Moreover, the intravenous injection of CIK cells promotes vascularization and accelerates diabetic wound healing. Therefore, CIK cells represent a novel, promising approach to treating diabetic wounds in clinical settings.

## Materials and methods

### Animals treatment

#### Diabetic mice and wound-healing model

The male C57BL/6J mice utilized in this experiment were obtained from the Guangdong Medical Lab Animal Center, China. To induce diabetes, the mice were injected intraperitoneally with a single dose of STZ (125 mg/kg, Sigma-Aldrich, St Louis, MO, USA) in citrate buffer (pH of 4.5). The *in vivo* hyperglycemic model was deemed successful if the glucose level of the tail vein blood consistently exceeded 16 mmol/l after 7 days. All diabetic mice have been utilized in the skin defect model investigation 1 week following the STZ treatment and randomly allocated into a control and a CIK groups. The mice were subjected to anesthesia by means of an intraperitoneal injection of 1% sodium pentobarbital at a dosage of 50 mg/kg. Subsequently, surgical scissors were employed to create full-thickness skin excisional wounds with a diameter of 8 mm on the dorsal trunk. Following this, the mice within the control group were subjected to tail vein injection of 0.1 ml phosphate buffer saline (PBS), whereas the animals in the CIK group had an injection of 0.1 ml PBS containing 2 × 10^6^ CIK cells. After the described treatments, the samples were analyzed using ImageJ software on Days 0, 3, 6 and 9, and the healing rate was indicated by the percentage of the initial wound area.

#### Histological analysis

Cervical dislocation was employed as the method of euthanasia for all mice, and the skin tissues were excised at the indicated time points. The samples underwent fixation in a 10% formalin solution for a duration of 24 h, followed by rinsing with running water, and dehydrated with anhydrous alcohol. The skin samples underwent sectioning into 5 μm paraffin sections utilizing a sledge microtome, followed by subsequent processing involving hematoxylin-eosin (H&E) and Masson’s trichrome staining. An optical microscope was employed to acquire all figures.

#### Immunohistochemical staining

For immunofluorescence staining, the paraffin sections of all samples were subjected to deparaffinization and antigen retrieval. This was achieved by heating the sections in a pH 6.0 citrate buffer at the specified time points, which were Days 3 and 6 as mentioned. The samples were subjected to incubation with the primary antibody for an extended period of time at a temperature of 4°C, following a prior washing step with PBS and blocked using bovine serum albumin (5%, 30 min). Following an additional washing step using PBS, the sections were subjected to incubation with the respective secondary antibodies in a light-restricted environment for an hour. We captured fluorescent images of the samples by using an inverted fluorescence microscope and randomly selected and evaluated 5–7 fields (400×) utilizing ImageJ software.

#### Dihydroethidium staining

The sections collected on Day 6 were frozen using liquid nitrogen and subjected to incubation in a light-restricted environment using dihydroethidium (DHE) (20 μM). Using inverted fluorescence microscopy, fluorescent images of the samples were obtained, and three fields (200×) underwent random selection. In addition, the fluorescence intensity of DHE was determined and analyzed using ImageJ.

#### RNA-sequencing and analysis

To conduct RNA-sequencing, three 1-cm-diameter samples nearby the healing wound were surgically removed on Day 6 from the control group and the CIK group. The isolation of total RNA was conducted utilizing the RNeasy Mini Kit (Qiagen, Germany). Following this, the concentration and quality of the RNA were assessed through a Qubit 3.0 fluorometer (Life Technologies, USA) and a NanoDrop One spectrophotometer (Thermo Fisher Scientific Inc., USA). The assessment of total RNA integrity was conducted via an Agilent 2100 bioanalyzer (Agilent Technologies Inc., USA). Only samples with RNA integrity values more than 7.0 were selected for the sequencing process. The RNA samples were prepared using one microgram of input material.

mRNA was subjected to differential expression analysis using the R package (version 3.4.3) edgeR. The differentially expressed RNA samples with a log2 FC value exceeding 1, *q* value below 0.05 and a mean FPKM surpassing 1 in one group, were selected for subsequent analysis as they were deemed to be significantly regulated. These selection criteria were used to maximize the analysis sensitivity, facilitating a comprehensive screening of potential candidate genes. These genes will next undergo validation using real-time PCR analysis on a larger sample set. To analyze biological processes, cellular components and molecular activities, we performed Kyoto Encyclopedia of Genes and Genomes (KEGG) pathway and gene ontology (GO) analyses using the EnrichR package (version 3.4.3) (http://www.genome.ad.jp/kegg).

#### Real-time PCR

The extraction of total RNA from each sample was performed as previously reported, followed by reverse transcription into cDNA in accordance with the guidelines provided by the manufacturer (DBI Bioscience, Germany). The ABI PRISM 7500 sequence detection system (ABI, Foster City, CA, USA) was employed to measure the mRNA expression levels of angiogenesis—vascular endothelial growth factor (*Vegf*) and platelet-derived growth factor (*Pdgf*); fibroblast activation: fibroblast growth factor (*Fgf*), transforming growth factor-β (*Tgf-β*) and *Collagen I*; inflammation: *Cd68*, *Il-1β*, *iNos2*, *Cd206*, *Il-4* and *Il-10*; and GAPDH. The GAPDH transcript levels were used as a reference gene for quantification. The primer sequences employed in the study are provided in Supplementary Table S1.

### *In vitro* experiment

#### Cultivation of cells

Human umbilical vein endothelial cells (HUVECs) obtained from American Type Culture Collection, USA and fibroblasts obtained from Chinese Academy of Sciences, Shanghai, China were grown in a 10% fetal bovine serum (FBS)—Dulbecco’s modified Eagle’s medium (DMEM) under conditions of a humidified incubator set at (37°C, 5% CO_2_). The isolation of peripheral blood mononuclear cells was performed using human peripheral blood obtained from individuals who were in good health through Ficoll density gradient centrifugation. These cells were multiplication-cultured according to the instructions of a CIK cytokine kit (RGL0030, Castd Regen-Geek, China) in a CIK cell serum-free medium (RGL0040, Castd Regen-Geek, China) in a humidified incubator (37°C, 5% CO_2_).

#### Preparation of CIK-CM

A CIK-conditioned medium (CM) was obtained by collecting the supernatants of CIK cells (10^6^ cells/well) cultivated in six-well plates with a volume of 2 ml/well following a 48-h incubation period. The CIK-CM was centrifuged and filter-sterilized (utilizing a 0.22 μm syringe filter) before being diluted at a proportion of 1:1 (v/v) via 10% FBS-DMEM as a 50% CM.

#### Viability assay of HUVECs and fibroblasts

AGEs were previously utilized as an *in vitro* model of the diabetic inflammatory condition [24]. HUVECs and fibroblasts were separately seeded in a 96-well plate at a primary density of 4000 cells per well and incubated for 24 h. The medium has been eliminated, and the HUVECs and fibroblasts were cultured under the following conditions:

1. Control group: cultivated within DMEM
2. CIK group: cultivated within CIK-CM
3. AGE group: cultivated within DMEM containing 100 μg/ml AGEs
4. CIK+AGE group: cultivated within CIK-CM containing 100 μg/ml AGEs

The cell proliferative capacity of both HUVEC and fibroblast groups was assessed using the cell counting kit-8 (CCK-8) assay.

#### Tube formation analysis

A 96-well plate containing (40 μl/well) of Matrigel was solidified in a humidified incubator (37°C, 5% CO_2_) for a duration of 2 h. Each HUVEC group (control, AGE, CIK and CIK+AGE) was seeded at a 1 × 10^4^ cells/well density under different conditions, as described above. After 12 h, a tube-like formation was observed, and subsequently, four distinct representative fields were captured in each well utilizing a microscope (100×). The total length of the tube of each group underwent quantitative evaluation employing ImageJ.

#### Scratch wound assays

The HUVECs were incubated at 5 × 10^5^ cells/well density in six-well plates until the formation of a confluent monolayer. The plates were washed with PBS after being scratched with a p-200 pipette tip to create an artificial incisional wound. The different mediums of each group (control, AGE, CIK and CIK+AGE) were introduced and cultured at a temperature of 37°C. After 12 h, cell migration was observed, and a total of four distinct representative fields were captured in all the wells utilizing a microscope (100×). The quantification of cell migration rate for each experimental group was conducted utilizing ImageJ.

#### Flow cytometry

CIK cells were obtained after multiplication culturing and evaluated with flow cytometry. The cells were treated with a 4% formaldehyde solution for fixation and subsequently permeabilized using 90% methanol. The anti-human antibodies for CD3 at 1/500 dilution (ab243873, Abcam, UK) and CD56 at 1/800 dilution (ab9272, Abcam, UK) were used as the primary antibodies, while DyLight^®^ 650-conjugated goat anti-rabbit IgG (ab96902, Abcam, UK) at 1/500 dilution and DyLight^®^ 488 goat anti-mouse IgG (ab96879, Abcam, UK) at 1/500 dilution were used as the secondary antibodies. FlowJo 10.0 software was utilized to analyze the data.

#### Western blotting

Different fibroblast groups were collected, and a BCA assay (Sigma-Aldrich, St Louis, MO, USA) was utilized to quantify the total protein. Subsequently, the proteins were subjected to sodium dodecyl sulfate–polyacrylamide gel electrophoresis to separate them based on their molecular weights. Following this, the separated proteins were then placed onto polyvinylidene fluoride membranes. The membranes were subjected to overnight incubation at a temperature of 4°C with one of the primary antibodies (BAX, BCL-2, Caspase 9 or C-caspase 3). Subsequently, the membranes were treated with secondary antibodies (Anti-rabbit IgG) at appropriate dilutions. The ChemiDicTM XRS+ imaging system (Bio-Rad, Hercules, CA, USA) was utilized to detect protein band densities. Subsequently, ImageJ was utilized to conduct a densitometric analysis of membranes.

### Materials

The DMEM, FBS and penicillin-streptomycin were acquired from Gibco (Grand Island, USA). Additionally, the trypsin-EDTA was obtained from Sigma-Aldrich (St Louis, USA). The primary antibodies against CD68 (ab283654), CD86 (ab239075), iNOS2 (ab283655), ARG (ab264066) and AGEs have been procured from Abcam (Cambridge, UK). PE anti-mouse CD86 (159204) and FITC anti-mouse CD206 (141703) have been collected from BioLegend (San Diego, USA). Alpha-smooth muscle actin (α-SMA) (BM0002) has been obtained from Boster Biological Technology (Wuhan, China). BCL-2 (AF6139), BAX (AF0120) and Caspase 9 (AF7022) antibodies were gathered from Affinity (Georgia, USA) and C-caspase 3 antibody has been procured from Bioss (Wuhan, China). Masson’s staining kit (G1340) was collected from Solarbio (Beijing, China).

## Results

### CIK cells promote healing of skin wound in diabetic mice

**Figure 1.**
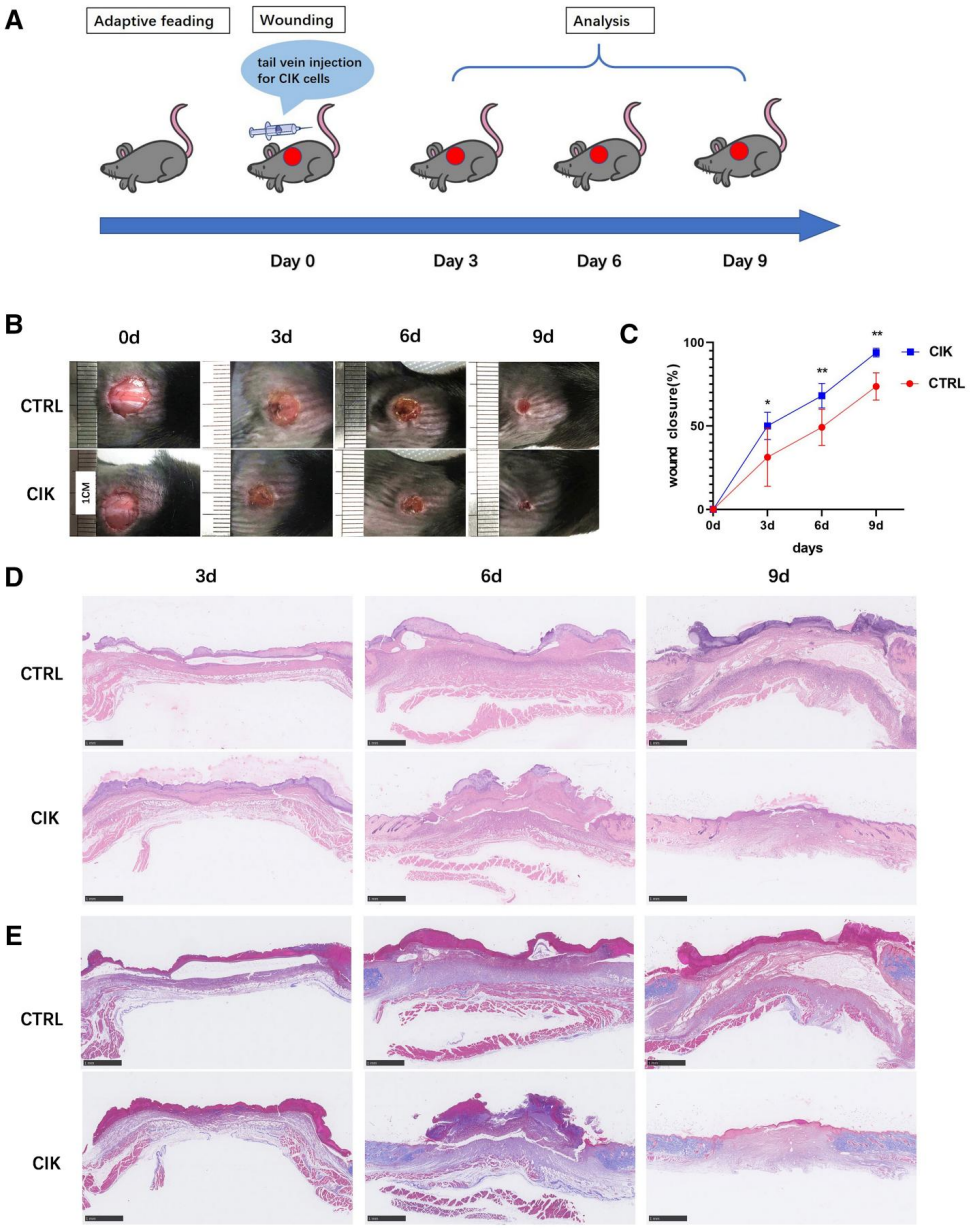
CIK cell treatment promotes diabetic wound healing in STZ-induced diabetic mice. (**A**) Schematic flowchart of the experiment conducted *in vivo*. (**B**) Representative images of wound closure at each time point. (**C**) Wound closure rates of each group were determined on Days 0, 3, 6 and 9 (one-way ANOVA). Data are expressed as mean ± SD. *n* = 6. *P < 0.05, **P < 0.01 contrasted to the control group. Error bars indicate SDs. (**D**) Representative H&E-stained pictures of wounds in various treatment groups on Days 3, 6 and 9; scale bar, 1 mm. (**E**) Masson’s trichrome staining of wound on Days 3, 6 and 9; scale bar, 1 mm.

Due to the properties of CIK cells, we investigated their treatment effects on diabetic cutaneous wound healing *in vivo*, where healing is typically delayed due to chronic inflammation. The mice underwent tail vein administration of CIK or PBS 1 week after the STZ treatment (Figure 1A). Compared to the mice treated with PBS, the CIK groups exhibited more significant closure of the wound areas 3–9 days after wounding, and the cutaneous defects in this group were mostly healed by Day 9 (Figure 1B and Supplementary Figure S1A). The quantified data indicated that the defect region of the CIK group exhibited a greater closure ratio compared with those of the control group at Days 3, 6 and 9 (Figure 1C and Supplementary Figure S1B). As shown through H&E staining, at each time point, the wound area across the CIK group was smaller than that present within the control group (Figure 1D). In the context of Masson’s trichrome staining, it was observed that the CIK group had a greater extent of collagen accumulation in comparison to the control group (Figure 1E and Supplementary Figure S2).

To ascertain the biosafety of CIK cell intravenous injection, we conducted *in vivo* evaluations of systemic toxicity using H&E staining experiments and serum biochemistry. Compared to healthy mice, the H&E staining results demonstrated no significant deterioration in the major organs of mice treated with CIK cell intravenous injection in Day 6 (Supplementary Figure S3A). Furthermore, the levels of alanine aminotransferase, aspartate aminotransferase, creatinine, creatine kinase and lactate dehydrogenase in CIK group showed no significant differences compared to the control group at 48 h after injection (Supplementary Figure S3B).

### CIK cells accelerate vascularization in diabetic wounds

**Figure 2.**
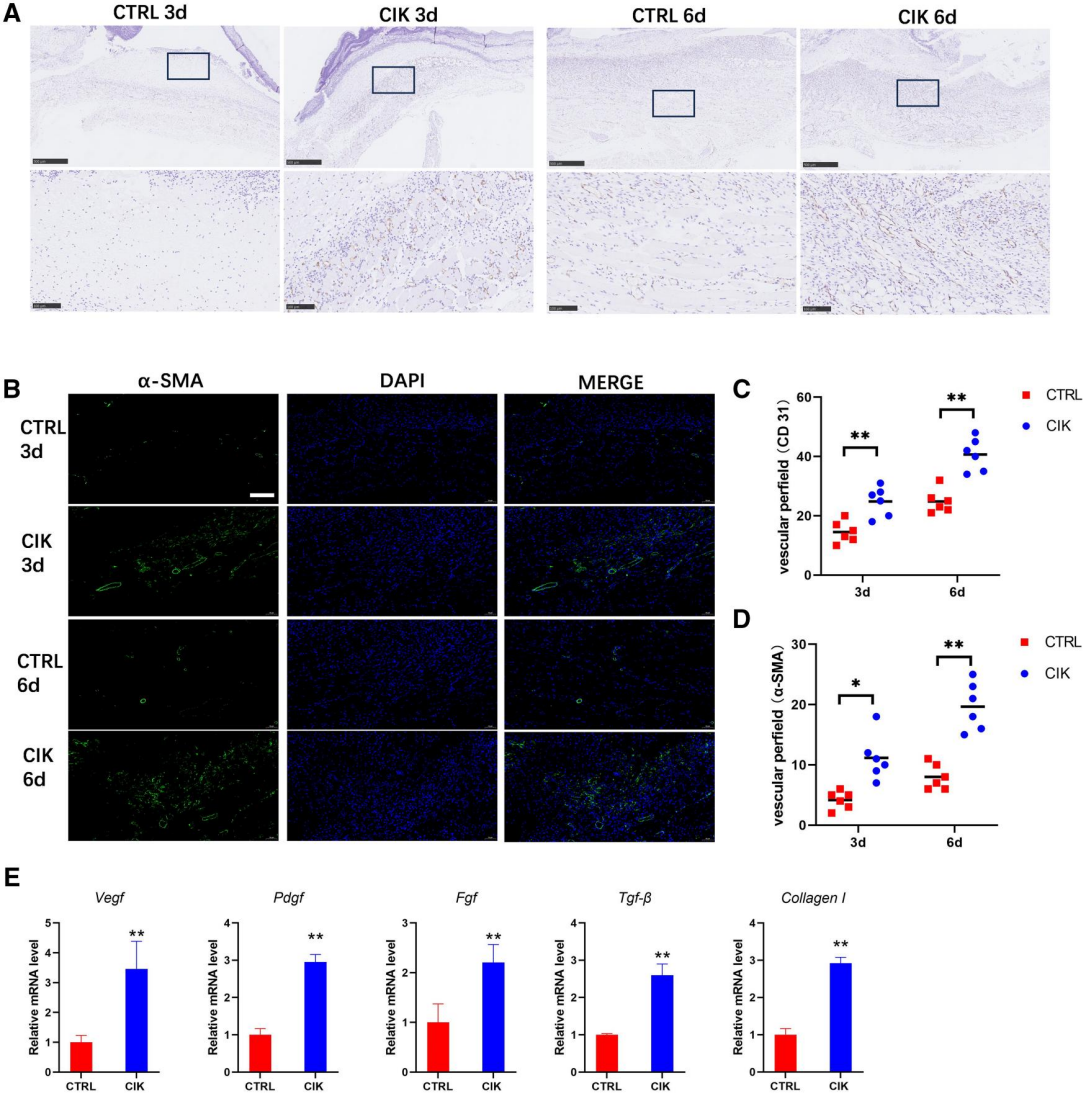
CIK cell treatment accelerates angiogenesis in diabetic wounds. (**A**) New blood vessels in healed diabetic wounds were observed on Days 3 and 6 through CD31 immumohistochemical staining on enlarged images; scale bar, 500 μm. The higher magnification images indicate a specific area within the black rectangular region marked in the upper image; scale bar, 100 μm. (**B**) New blood vessels in healed diabetic wounds were observed on Days 3 and 6 through α-SMA immunofluorescent staining on enlarged images; scale bar, 100 μm. (**C**) The quantification of the quantity of newly formed blood vessels (CD31, one-way ANOVA). **P < 0.01 in comparison to the control group. *n* = 6. (**D**) Quantification of the number of new blood vessels (α-SMA, one-way ANOVA). *P < 0.05, **P < 0.01 compared to the control group. *n* = 6. (**E**) Real-time PCR analysis of VEGF, PDGF, FGF, TGF-β and collagen I has been conducted on Day 6 (one-way ANOVA). **P < 0.01 contrasted with the control group. *n* = 3. Error bars indicate SDs.

Tissue regeneration patterns, especially the spatial distribution of neovascular networks, are closely related with angiogenic patterns during the early wound-healing process [25]. Wound repairing and angiogenic processes are highly interdependent in a hyperglycemic microenvironment [26]. We used immunofluorescent staining for α-SMA and immunohistochemical staining for CD31 on Days 3 and 6 to indicate newly formed capillaries in the initial stage of wound healing (Figure 2A and B). The results of the quantitative analysis of the CIK treatment demonstrated a significantly elevated number of positive CD31 and α-SMA staining compared to the control group (Figure 2C and D). Furthermore, on Day 6, the levels of mRNA for factors associated with angiogenesis (*Vegf* and *Pdgf)* and fibroblast activation (*Fgf, Tgf-β* and *Collagen I*) were significantly higher within the post-traumatic diabetic mice treated with CIK than in the control group (Figure 2E). The aforementioned data, along with the previous findings, suggest that CIK cells increase the number of newly formed capillaries and promote the proliferation of fibroblasts by upregulating the abovementioned regeneration factors.

### CIK cells reduce inflammatory response during wound healing

**Figure 3.**
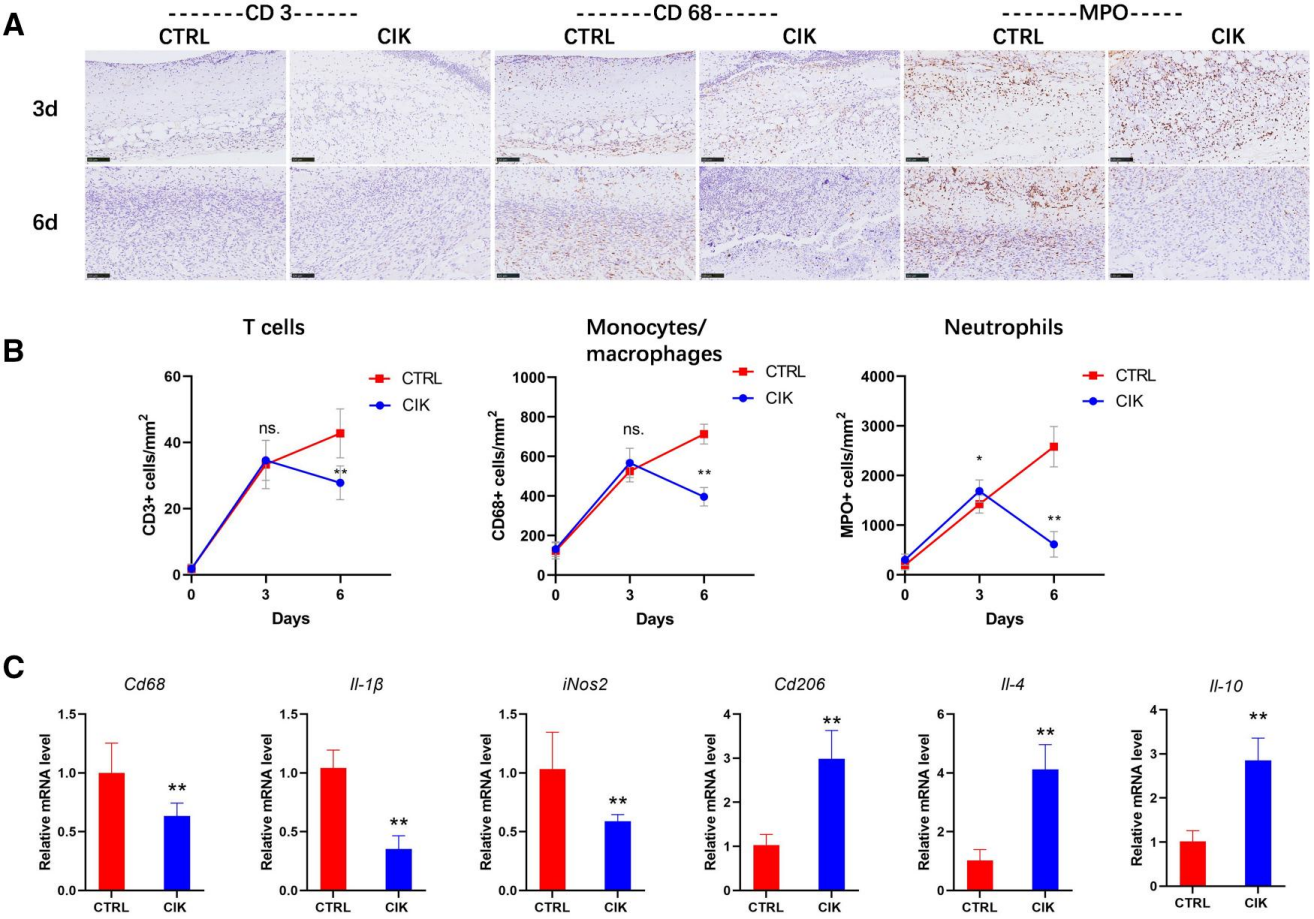
Impact of CIK cells on the inflammation of diabetic wounds. (**A**) Representative pictures of recruitment of immune cells during the diabetic wound-healing process in skin tissues. MPO: myeloperoxidase. (**B**) Quantification of specific immune cell recruitment on Days 3 and 6. *P < 0.05, **P < 0.01 by unpaired *t*-test. Error bars indicate SDs. *n* = 3. (**C**) Real-time PCR analysis of CD86, IL-1β, iNOS2, CD206, IL-4 and IL-10 has been conducted on Day 6 (one-way ANOVA). **P < 0.01 contrasted with the control group. *n* = 3. Error bars indicate SDs.

We analyzed the recruitment of immune cells during the wound-healing process on Days 3 and 6 through histological and quantitative evaluation. In the context of post-traumatic inflammatory patterning in the diabetic hyperglycemia environment, the results showed a remarkably upregulated activation of the inflammation response on Day 3 and a downregulated activation of the recruitment of immune cells, including T cells, monocytes/macrophages and neutrophils, by Day 6 in the CIK group; conversely, inflammation continued to increase within the control group (Figure 3A and B). In addition, the quantification of mRNA levels was evaluated using RT-PCR. According to the results, after Day 6, the pro-inflammatory cytokines (*Cd68, Il-1β* and *iNos2*) were markedly decreased, while the anti-inflammatory cytokines (*Cd206, Il-4* and *Il-10*) were markedly overexpressed in the CIK group contrasted with the control group (Figure 3C).

### CIK cells induce anti-inflammatory polarization of macrophages

**Figure 4.**
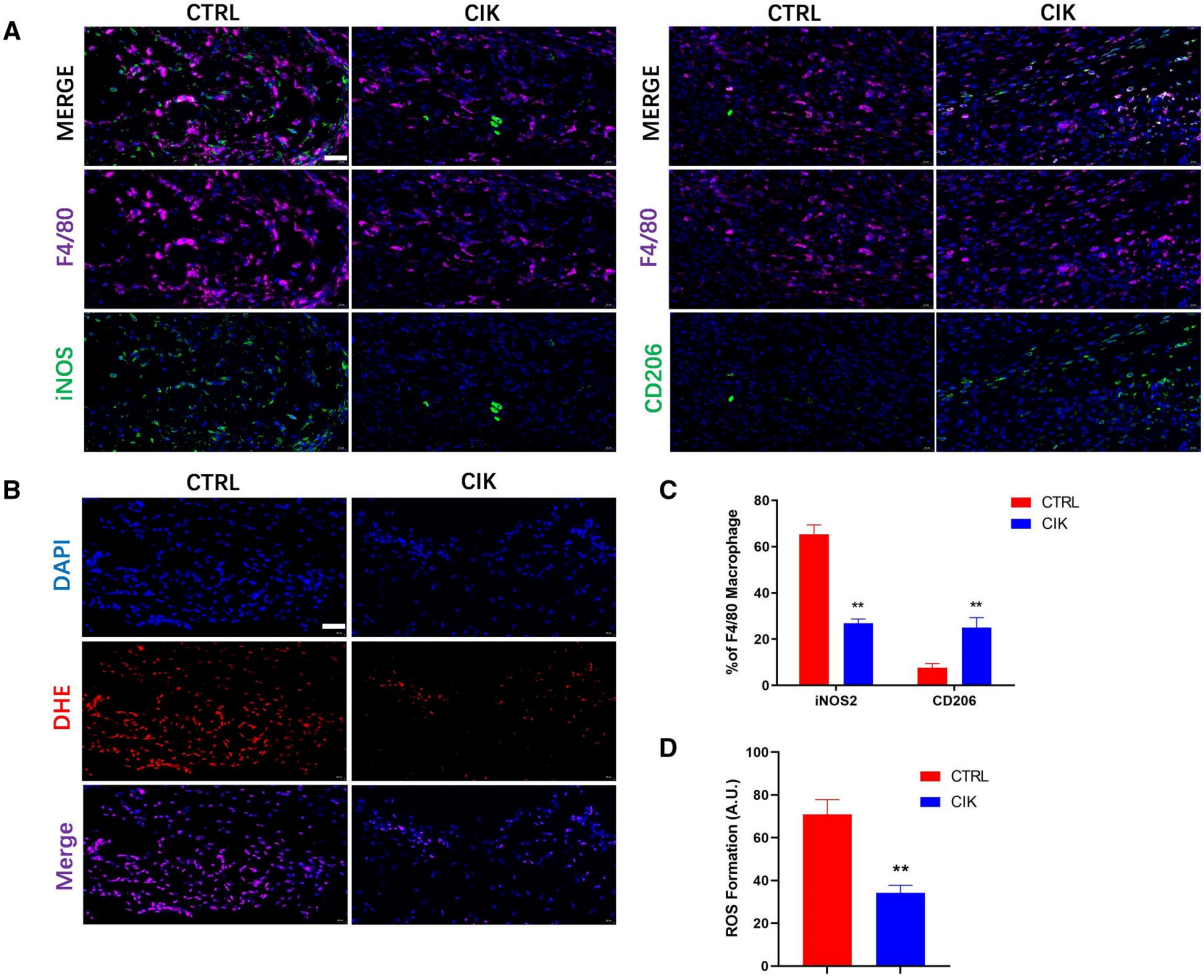
CIK cells induce polarization of macrophages in diabetic wounds. (**A**) Dual immunofluorescence staining has been carried out on wound tissues with F4/80 and iNOS or CD206 on Day 6; scale bar, 40 μm. (**B**) DHE staining determined the extent of ROS production in diabetic wounds on Day 6; scale bar, 40 μm. (**C**) Respective cells from the immunofluorescence staining pictures and the percentage of iNOS+/F4/80+ (M1 polarized) and CD206 +/F4/80+ (M2 polarized) cells per microscopic field (one-way ANOVA). **P < 0.01 contrasted to the control group. *n* = 6. Error bars indicate SDs. (**D**) DHE fluorescence intensity was analyzed (one-way ANOVA). **P < 0.01 contrasted to the control group. *n* = 6. Error bars indicate SDs.

Based on the aforementioned results of the inflammatory response, the difference between the CIK group and the control group was observed to be most significant on the sixth day. Therefore, we conducted an analysis of the macrophage polarization process on Day 6. The results of immunofluorescence staining showed that the M1-type macrophage marker iNOS2 was more downregulated within the CIK group than within the control group at Day 6, whereby the M2-type macrophage indicator ARG was more upregulated in the CIK group (Figure 4A and C). DHE staining has been conducted on Day 6 after surgery and revealed significantly decreased reactive oxygen species (ROS) formation within the CIK group as contrasted with the control group (Figure 4B and D). Thus, evidently, CIK cells can induce the polarization of M2-type macrophages, consequently decreasing ROS formation and the inflammatory response.

### CIK cells alleviate inflammation and promote cell proliferation

**Figure 5.**
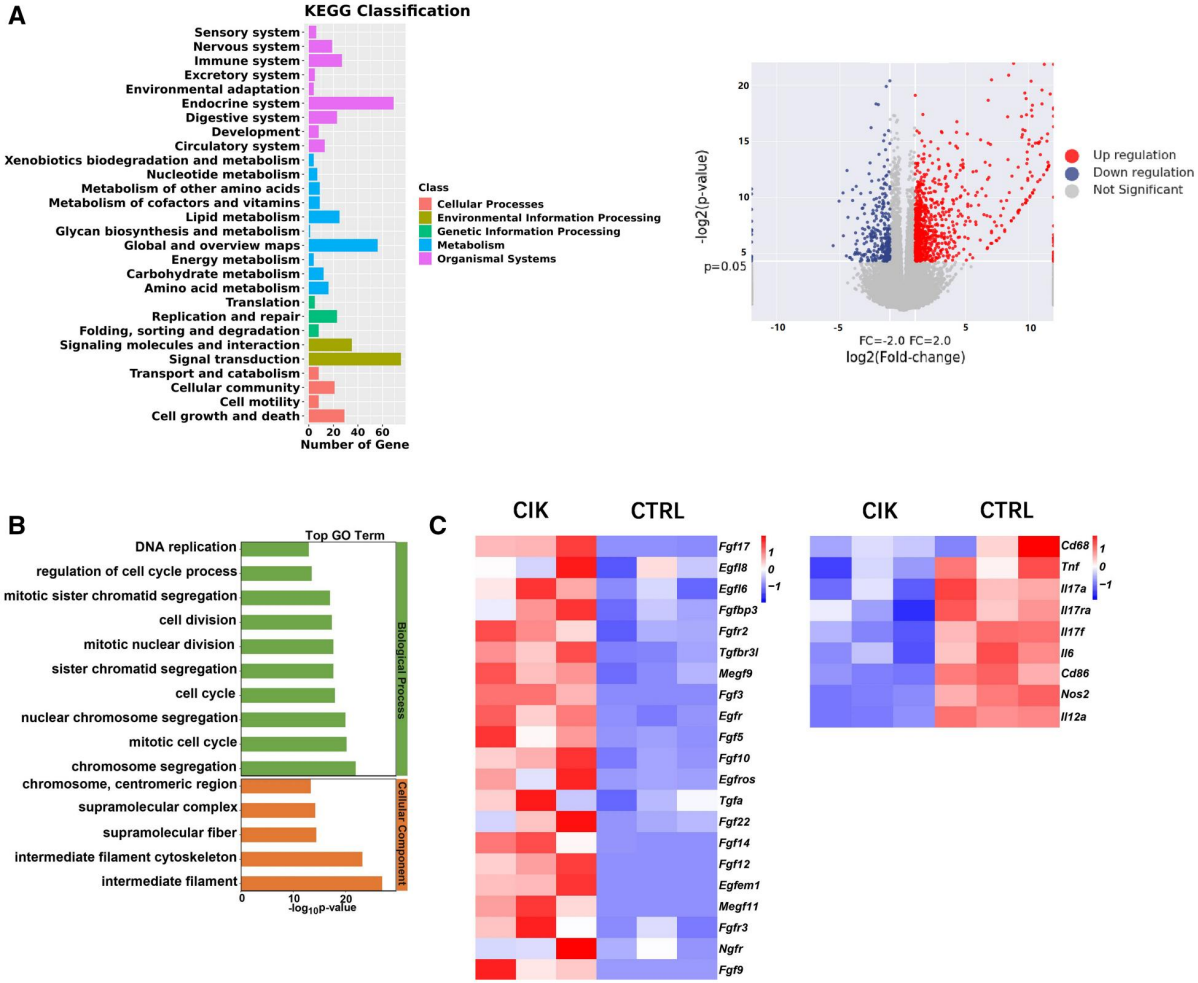
Effect of CIK cell treatment on diabetic wounds. (**A**) KEGG classification and volcano plots represent variations in gene expression among the CIK and control groups. (**B**) Enriched biological process of GO analysis. (**C**) Results of heat map of the RNA-seq analysis displays relative expression of growth factor-and immunity-related gene transcripts on Day 6.

Target genes were used for KEGG pathway enrichment and GO clustering analysis. The findings obtained from the KEGG differential genes pathway analysis indicated that the target genes were mainly enriched in the endocrine system for cellular processes; global and overview maps for metabolism; signal transduction, signaling molecules and the interaction of signaling molecules for the purpose of environmental information processing; replication and repair for the processing of genetic information; cell growth and death; and cellular community for organismal systems (Figure 5A). Furthermore, the GO analysis findings revealed that most of the differential genes were positively correlated with intermediate filaments in the cytoskeleton, chromosome segregation, the mitotic cell cycle and other cell proliferation-related processes (Figure 5B). The RNA-seq data in the gene expression profile revealed that growth factor-related genes, such as *Fgf*, epidermal growth factor (*Egf*) and *Tgf*, were significantly active in the CIK group 6 days after surgery, whereas inflammation-related genes, such as *Cd68*, *Tnf, Il-17*, *Il-6*, *Cd86*, *Nos2* and *Il-2a*, were markedly downregulated (Figure 5C).

### CIK cells facilitate cell proliferation and activation in a hyperglycemic environment

**Figure 6.**
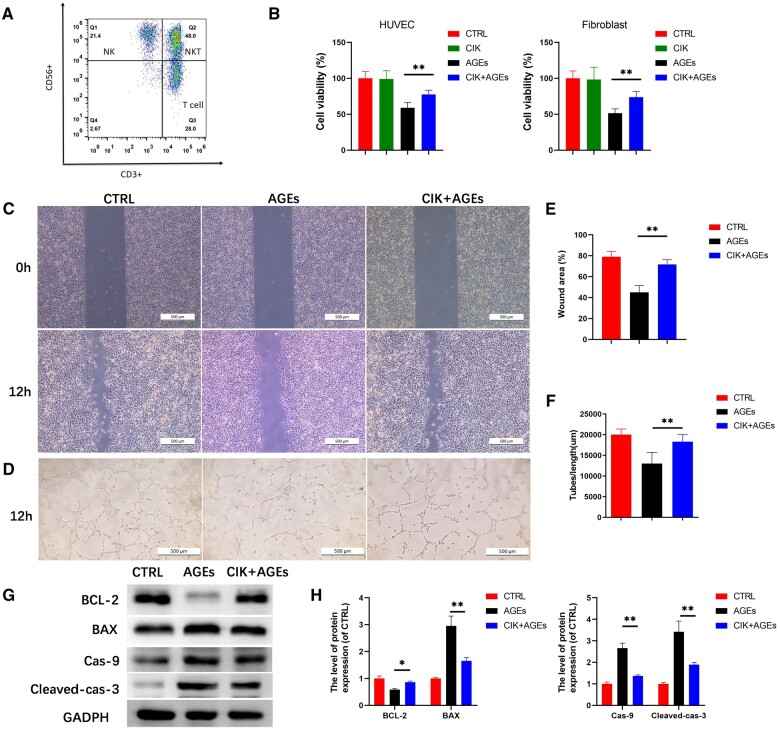
CIK cells decrease AGE-induced apoptosis in HUVECs. (**A**) CIK cells were evaluated with flow cytometry. (**B**) The results of CCK-8 on HUVECs and fibroblasts for a duration of 24 h (one-way ANOVA). Error bars indicate SDs. *n* = 6. (**C**) Scratch wound assays were performed on different groups of HUVECs for 12 h; scale bar, 500 μm. (**D**) Tube formation assays were performed on different groups of HUVECs for 12 h; scale bar, 500 μm. (**E**) Scratch areas have been measured through image analysis (one-way ANOVA) **P < 0.01 contrasted to the AGE group; *n* = 6; error bars indicate SDs. (**F**) The tube lengths have been determined through image analysis (one-way ANOVA). **P < 0.01 compared to the AGE group. *n* = 6. Error bars indicate SDs. (**G** and **H**) Protein expression levels of BAX, BCL-2, Caspase-9, Cleaved caspase-3 and GAPDH in fibroblasts of every treated group, as previously outlined. **P < 0.01 or *P < 0.05 contrasted to the AGE group. *n* = 3. Error bars indicate SDs.

CIK cells were successfully generated and proliferated *in vitro* to achieve a CIK cell [27] percentage that yielded a CD3^+^CD56^+^value of 46% (Figure 6A). To assess the effect of CIK cells in the AGE microenvironment, we used a CCK-8 assay to analyze the performance of HUVECs and fibroblasts indirectly co-cultured with CIK cells. The results showed that the HUVECs and fibroblasts cultured in CIK-CM (the CIK group) did not proliferate to the extent of HUVECs and fibroblasts cultured in DMEM (the control group). However, the percentage of HUVECs and fibroblasts was greater when cultured in CIK-CM containing AGEs (the CIK+AGE group) than when cultured in DMEM containing AGEs (the AGE group) (Figure 6B).

Tube formation and scratch wound assays have been utilized to investigate the efficacy of CIK-CM in AGE-treated HUVECs. Representative photomicrographs revealed cell migration structures (Figure 6C) and capillary-like structures (Figure 6D). Quantitative area measurement revealed that the AGE treatment (the AGE group) suppressed the migration ability of HUVECs and that CIK-CM effectively restored this cell viability (Figure 6E). The quantitative measurement of tube lengths demonstrated that the AGE treatment markedly decreased the quantity of capillary-like structures, whereas the CIK-CM intervention restored angiogenesis ability (Figure 6F).

Additionally, we evaluated the levels of expression of the following apoptosis-associated proteins: BAX, BCL-2, Caspase-9 and Cleaved caspase-3. The level of BCL-2 expression in AGE-treated fibroblasts was observed to be underexpressed, while those of BAX, Caspase-9 and Cleaved caspase-3 were upregulated. However, after CIK cell treatment, the expression level of BCL-2 was overexpressed contrasted with that in the AGE group. In contrast, BAX, Caspase-9 and Cleaved caspase-3 expression levels have been reduced relative to those in the AGE group (Figure 6G and H).

## Discussion

An inflammatory post-traumatic environment affects diabetic cutaneous wound healing, repair and regeneration [28] through cytokines’ paracrine signaling between immune cell populations; it also impacts skin regeneration [29]. Hyperglycemia-induced excessive inflammation and disordering of the inflammatory environment leads to delayed tissue repair [30]. It is well known that suitable inflammatory patterning promotes initial angiogenesis and fibroblast generation, enhancing the synthesis of collagen and, subsequently, facilitating wound closure [31]. Our research demonstrated that CIK cells facilitate vascularization and diabetic skin wound repair through their effect on inflammatory regulation. This approach restores immune homeostasis with a CIK cell-based treatment and is a novel therapeutic method for diabetic wound healing with the potential to resolve disordered inflammation patterning and provide regeneration factors.

CIK cell subsets—namely NK, NKT and T cells—are of particular interest due to their potential clinical effects [32] and easy proliferation through straightforward, inexpensive protocols [33]. The strategies adopted to accelerate diabetic wound healing by restoring immune homeostasis focus on resolving persistent inflammation and generating cell factors [9]. The basic research and clinical investigations associated with CIK cells have validated their safety and possibility for malignant diseases treatment [21]. However, no previous investigations have reported the potential function of CIK cells in diabetic wound repairing and in the resolution of a post-traumatic diabetic inflammatory microenvironment. CIK cells secrete immune function-related cytokines [34] and increase the expression level of inflammatory regulation-related genes while under stimulation [35]. Our study validates the hypothesis that CIK cells can actively modulate the post-traumatic immune homeostasis and inflammatory pattern in diabetic wounds, leading to increased expression levels of cytokines (EGF, TGF-β and FGF), the provision of growth factors (VEGF and PDGF) and enhanced cell proliferation.

Recent research has revealed various functions of NK cells, such as resolving inflammation and modulating the antigen-presenting cell function [36]. Mice deficient in NKT cells have been shown to produce lower concentrations of IL-4 and IL-10 but substantially increased amounts of IFN-γ [19]. In our study, the CIK cells could reduce anti-inflammatory cytokines expression level and resolve the problem of recruiting immune cells, such as T cells, monocytes/macrophages and neutrophils, at Day 6 after treatment. However, the post-traumatic acute inflammatory phase (Day 3) was not significantly suppressed by these cells. Appropriate pro-inflammatory responses have been shown to be the optimal inflammation patterning for tissue regeneration [37], as opposed to the sustained, excessive inflammatory microenvironment resulting from disrupted diabetic homeostasis. Although the specific influence of CIK cells on diabetic skin during the post-traumatic acute inflammation phase remains unclear, this mechanism may be associated with the secretion of pro-inflammatory cytokines via NK cells. Moreover, NK cells secrete granzyme B and perforin to induce cell apoptosis after being stimulated, and relevant pro-inflammatory cytokines, including TNF-α and IFN-γ, to further the immune response [38]. An important function of IFN-γ in the wound-healing process is to stimulate monocytes/macrophages to enhance VEGF production and promote vascularization [39]. Meanwhile, NKT cells increase the expression level of anti-inflammatory mediators, eliminate excessive inflammation in the tissue microenvironment and re-establish the natural course of the inflammation stages. Based on the functions of the abovementioned cells, the inflammatory patterning restored by the CIK cell population in the diabetic microenvironment appears more suitable for skin tissue repair.

Macrophages are critical to the regulation of inflammatory patterning and are characterized by diversity and plasticity, switching phenotypes as the microenvironment changes during the wound-healing process [40]. The complicated interactions among vascular endothelial cells, epithelial cells, fibroblasts and inflammation-related cells affect skin tissue regeneration [41]. The macrophage phenotype can be polarized to two extremes, with pro-inflammatory (M1 polarized) phenotypes activated via IFN-γ and LPS [42] and anti-inflammatory (M2 polarized) phenotypes activated via IL-4 or IL-10 [43]. According to our current research data, CIK cell treatment has specific anti-inflammatory therapeutic effects in the post-traumatic diabetic inflammatory environment, as it decreases the polarization of M1-type macrophage phenotypes and increases the polarization of M2-type macrophage phenotypes. Several cytokines were reported to produce a potent pro-inflammatory impact [7], such as TNF, NOS_2_, CD86 and CD68. These cytokines are reduced at Day 6, which is in agreement with the ratio variation trend of M1/M2 polarization. Similarly, these data are consistent with the results achieved for immune cell recruitment and ROS accumulation. Taken together, our results suggest that CIK cells exhibit a clear anti-inflammatory effect and promote diabetic wound closure.

A primary mechanism contributing to non-healing diabetic wounds is the build-up of AGEs in a hyperglycemic microenvironment, which compromises cell activity, induces excess inflammation and causes the pathogenesis of diabetic skin tissue [44]. AGEs disrupt both extracellular and intracellular structure and function [45], resulting in impaired vascularization. Our result demonstrates that the CM of CIK cells can restore angiogenesis and the migration ability of HUVECs in the AGE environment and suggests that CIK cells restore the cellular function of vascular endothelial cells through paracrine action.

Growth factors are key to activating molecular events, which explains the promoted skin tissue regeneration in diabetic wounds. In this study, the results of RNA-sequencing demonstrate that CIK treatment increases the expression of growth factors, which are the prime cutaneous tissue for efficient and accelerated wound healing. It has been well reported that TGF-β causes extracellular matrix contraction, resulting in wound closure by promoting myofibroblast production from fibroblasts, and that it increases collagen synthesis [46]. According to some previous experimental results, TGF-β production was decreased in NKT cell-deficient mice, suggesting that the reduction of collagen deposition and myofibroblasts is associated with the downregulation of NKT cells [20]. Furthermore, NK cells’ capacity for producing IL-22 merits attention [47]. Moreover, IL-22 can significantly accelerate diabetic wound closure by promoting re-epithelialization, angiogenesis and granulation tissue formation [48], also suggesting the therapeutic potential of NK cells in chronic refractory diabetic wounds.

In this study, we successfully isolated and multiplication-cultured CIK cells, providing a novel cell type for diabetic wound therapy. CIK cells appear promising for clinical cell therapy as an accurate, proactive immunomodulation strategy. Numerous clinical studies of this therapeutic strategy were published, demonstrating its safety and efficacy in treating cancer individuals [49]. However, if CIK cells are to be adopted as therapeutic agents in clinical use, several limitations must be considered, such as the widespread, large-scale preparation of CIK cells. Resolving these limitations can contribute to promote the clinical application of CIK cells and to evaluate the clinical response. Since most of the clinical trials are still in an early phase [50], the immunoregulatory role of CIK cells in the inflammation and regeneration of diabetic wound should be evaluate in depth. The discovery that CIK cells serve as upstream regulators of macrophage function [15] offers a novel cellular target for addressing chronic inflammation. Exploring the interaction between CIK cells and innate immunocytes may reveal essential molecular regulators capable of resolving chronic inflammation and reinstating immune homeostasis. The utilization of CIK cells as a routine treatment for diabetic wounds in clinical practice requires further large-scale multicenter trials based on these cells.

## Conclusion

CIK cells possess significant benefits in restoring the homeostasis of the cellular microenvironment. The precise and proactive immunomodulation can switch macrophage polarization tendencies and provide several cell growth factors in post-injury diabetic wounds. These results indicate that CIK cells are an effective therapy for diabetic wound repair and hold great promise for clinical curative effects.

## Supplementary Material

rbad116_Supplementary_Data

## Data Availability

The article and/or Supplementary Materials contain all the accessible data.
