# Supplementary material for: Harnessing cytokine-induced killer cells to accelerate diabetic wound healing: an approach to regulating post-traumatic inflammation
Source: Regen Biomater. 2024 Jan 9;11:rbad116. doi: 10.1093/rb/rbad116 (PMC10850840; doi:10.1093/rb/rbad116)
Supplement: rbad116_Supplementary_Data [file rbad116_supplementary_data.zip › Supplementary Data.docx]

**Harnessing Cytokine-Induced Killer Cells to Accelerate Diabetic Wound Healing: A Novel Approach to Regulate Post-traumatic Inflammation**

Yixi Yang^1, #^, Cheng Zhang^1, #^, Yuan Jiang^1, #^, Yijun He^2^, Jiawei Cai^1^, Lin Liang^1^, Zhaohuan Chen^1^, Sicheng Pan^1^, Chu Hua^1^, Keke Wu^1, *^, Le Wang^1, *^, Zhi-Yong Zhang^1, *^

^#^: these authors contributed equally to this work.

^*^: co-corresponding authors.

1. Translational Research Centre of Regenerative Medicine and 3D Printing of Guangzhou Medical University, Guangdong Province Engineering Research Center for Biomedical Engineering, State Key Laboratory of Respiratory Disease, Department of Orthopaedic Surgery, Medical Technology and Related Equipment Research for Spinal Injury Treatment, City Key Laboratory, The Third Affiliated Hospital of Guangzhou Medical University, School of Biomedical Engineering, Guangzhou medical University, Guangzhou, Guangdong, 510150, P. R. China
2. Department of Osteoarthropathy and Sports Medicine, Panyu Central Hospital, Guangzhou, 511400, P. R. China

E-mail addresses: drzhiyong_pub@outlook.com (Prof. Zhi-Yong Zhang), drwukeke@126.com (Prof. Keke Wu), wangle8273@163.com (Prof. Le Wang)

**Supplementary Data**

|  | Gene | Primer sequence（5’-3’） | | Amplification length（bp） |
| --- | --- | --- | --- | --- |
| 1 | r-GAPDH | Forward： | ATGTGTCCGTCGTGGATCTG | 142 |
|  |  | Reverse： | AAGTCGCAGGAGACAACCTG |  |
| 2 | r-VEGF | Forward： | AACGATGAAGCCCTGGAGTG | 117 |
|  |  | Reverse： | GCTGTGCTGTAGGAAGCTCA |  |
| 3 | r-PDGF | Forward： | ATTAAGCCGGTCCCAACCTG | 169 |
|  |  | Reverse： | AATGGGACCTGACTTGGTGC |  |
| 4 | r-FGF | Forward： | CCGTGGCAGTTGGAATTGTG | 175 |
|  |  | Reverse： | CCGCTGTGTGTCCATTTAGC |  |
| 5 | r-TGF-β | Forward： | CACTCCCGTGGCTTCTAGTG | 142 |
|  |  | Reverse： | CTGGCGAGCCTTAGTTTGGA |  |
| 6 | r-collgen I | Forward： | TTCTCCTGGCAAAGACGGAC | 198 |
|  |  | Reverse： | CGGCCACCATCTTGAGACTT |  |
| 7 | r-IL-1β | Forward： | AACCTTTGACCTGGGCTGTC | 144 |
|  |  | Reverse： | AAGGTCCACGGGAAAGACAC |  |
| 8 | r-CD68 | Forward： | CCGTTACTCTCCTGCCATCC | 156 |
|  |  | Reverse： | TGGTGGCAGGGTTATGAGTG |  |
| 9 | r-iNOS2 | Forward： | GCTATGGCCGCTTTGATGTG | 184 |
|  |  | Reverse： | ACCTCCAGTAGCATGTTGGC |  |
| 10 | r-IL-10 | Forward： | TGCTGCCTGCTCTTACTGAC | 106 |
|  |  | Reverse： | CTAGGAGCATGTGGCTCTGG |  |
| 11 | r-CD206 | Forward： | AAAACTGACTGGGCTTCCGT | 177 |
|  |  | Reverse： | TCTGCTCCACAATCCCGAAC |  |
| 12 | r-IL-4ra | Forward： | GTAGGGCTTCCAAGGTGCTT | 117 |
|  |  | Reverse： | GGCATCGAAAAGCCCGAAAG |  |
|  |  | Reverse： | ACCTCCAGTAGCATGTTGGC |  |

**Table S1.** Mice primer sequences used in real-time PCR analysis.


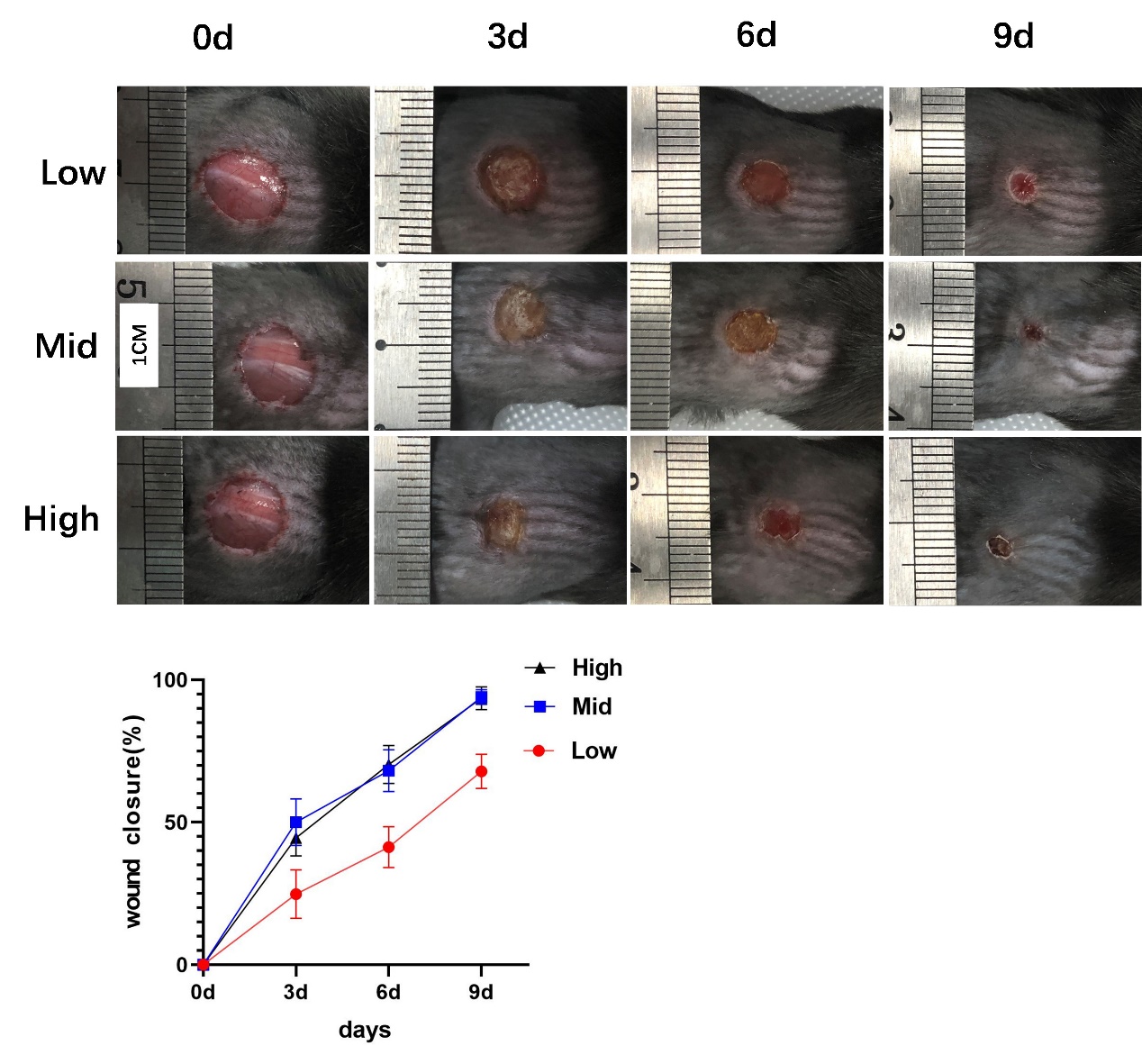


**Figure S1.** Different quantity of CIK cells treatment promotes diabetic wound healing in STZ-induced diabetic mice. (A) Representative images for different quantity of CIK cells treatment on diabetic wound closure. Low: 5x105 CIK cells; Mid: 2x106 CIK cells; High：5x106 CIK cells (B) Wound closure rates of each groups were determined on Days 0, 3, 6 and 9 (one-way ANOVA). Data were presented as the means ± SD. n = 6. Error bars represent SDs


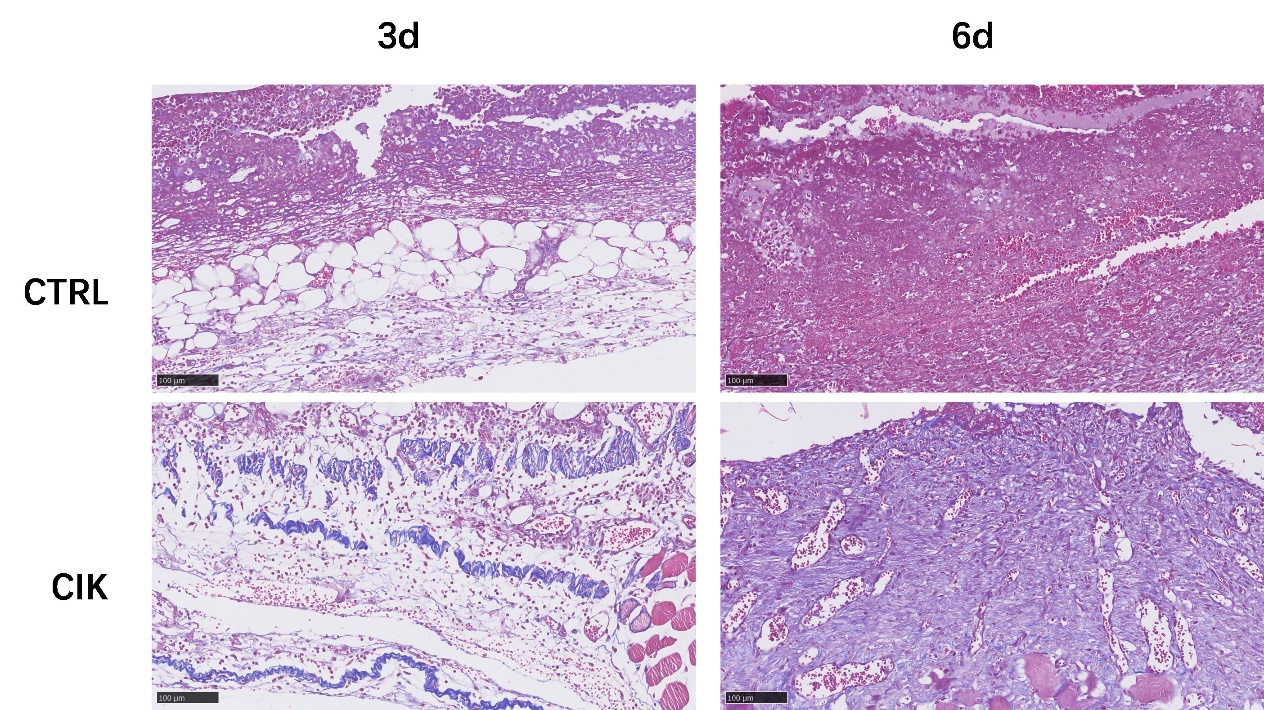


**Figure S2.** Higher magnification images of Masson's trichrome-stained tissues (blue for collagen). Scale bars, 100 μm.


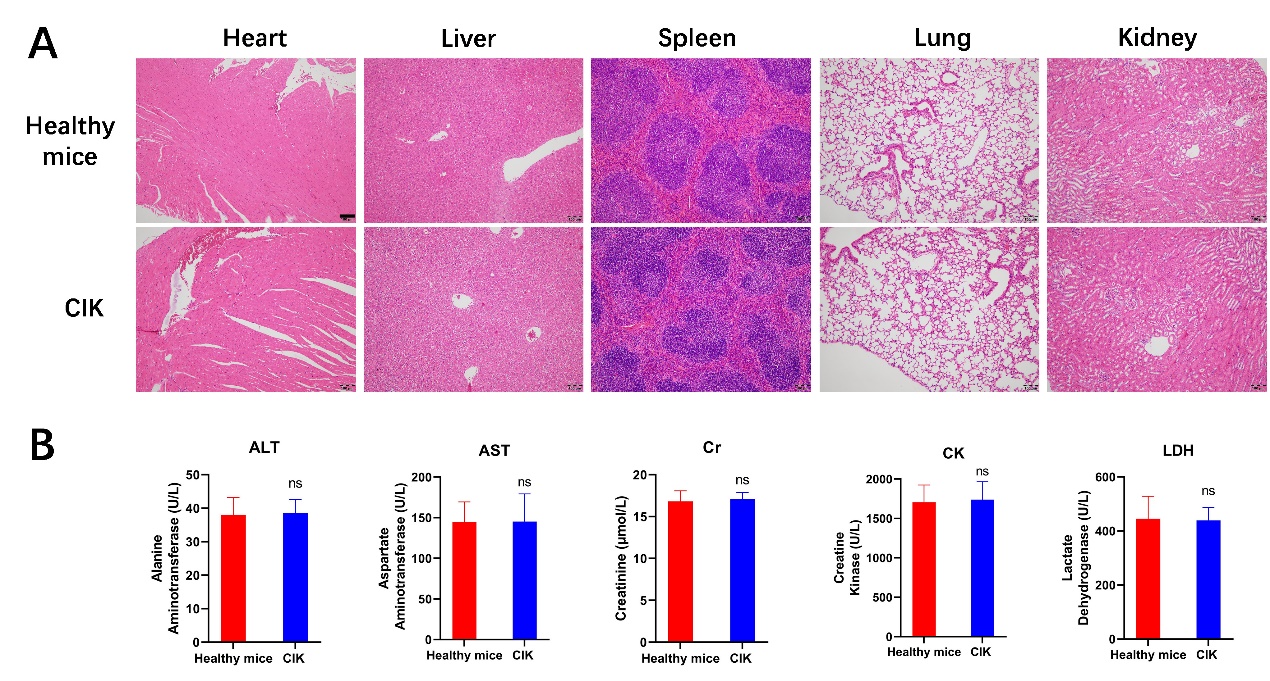


**Figure S3.** (A) Representative H&E-stained pictures of tissue sections; scale bar, 100μm. (B) Blood biochemistry analysis of mice at 48 h after injection, n=3.
