# Supplementary figures and images for: Harnessing cytokine-induced killer cells to accelerate diabetic wound healing: an approach to regulating post-traumatic inflammation
Source: Regen Biomater. 2024 Jan 9;11:rbad116. doi: 10.1093/rb/rbad116 (PMC10850840; doi:10.1093/rb/rbad116)

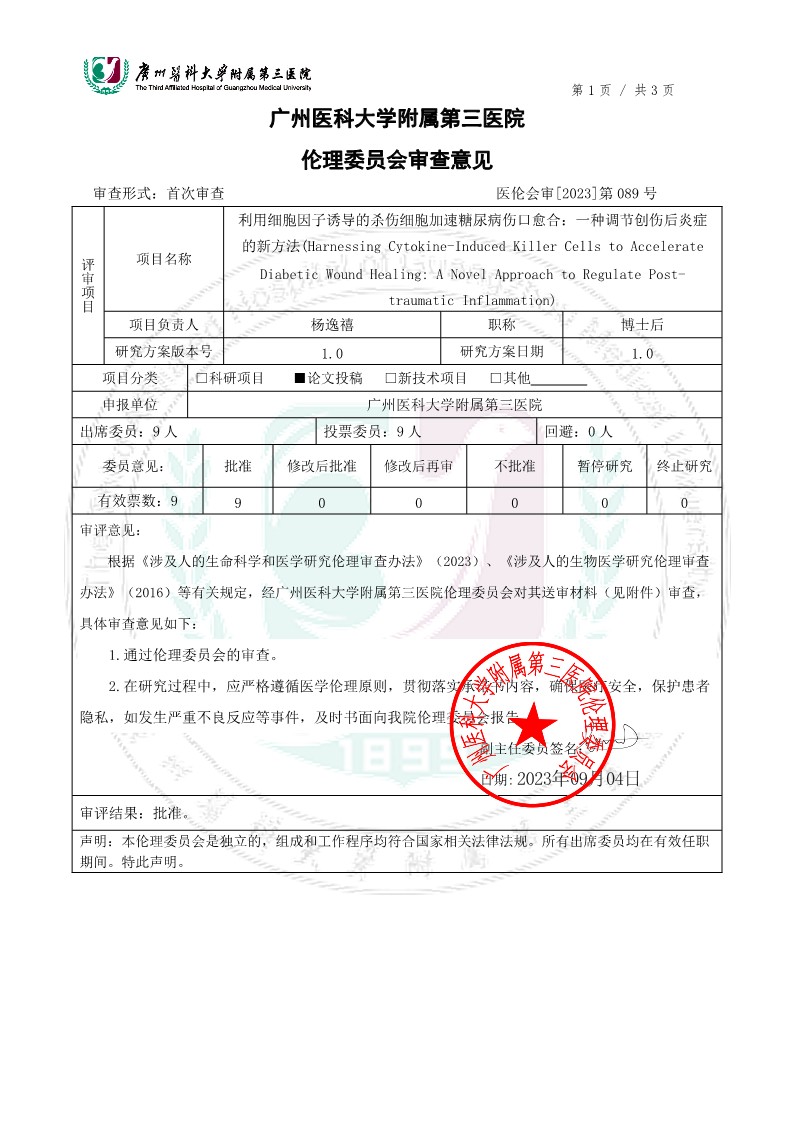

Supplement: rbad116_Supplementary_Data [file rbad116_supplementary_data.zip › ethic committee.jpg]
